# Reduced Parasympathetic Reactivation during Recovery from Exercise in Myalgic Encephalomyelitis/Chronic Fatigue Syndrome

**DOI:** 10.3390/jcm10194527

**Published:** 2021-09-30

**Authors:** Jessica Van Oosterwijck, Uros Marusic, Inge De Wandele, Mira Meeus, Lorna Paul, Luc Lambrecht, Greta Moorkens, Lieven Danneels, Jo Nijs

**Affiliations:** 1 Departments of Physiotherapy, Human Physiology and Anatomy, Vrije Universiteit Brussel, 1090 Brussels, Belgium; jo.nijs@vub.ac.be; 2 Spine, Head and Pain Research Unit Ghent, Department of Rehabilitation Sciences, Faculty of Medicine and Health Sciences, Ghent University, Campus UZ Ghent, Corneel Heymanslaan 10, B3, 9000 Ghent, Belgium; Inge.DeWandele@UGent.be (I.D.W.); mira.meeus@ugent.be (M.M.); lieven.danneels@ugent.be (L.D.); 3 Institute for Kinesiology Research, Science and Research Centre Koper, 6000 Koper, Slovenia; uros.marusic@zrs-kp.si; 4 Department of Health Sciences, Alma Mater Europaea—ECM, 2000 Maribor, Slovenia; 5 Department of Rehabilitation Sciences and Physiotherapy, Faculty of Medicine and Health Sciences, University of Antwerp, 2610 Antwerp, Belgium; 6 Nursing and Health Care, School of Medicine, University of Glasgow, Glasgow G12 8LL, UK; Lorna.Paul@glasgow.ac.uk; 7 Medical Private Practice for Internal Medicine, 9000 Ghent, Belgium; Lambrechtlj@skynet.be; 8 Department of Internal Medicine, University Hospital Antwerp (UZA), 2650 Antwerp, Belgium; Greta.Moorkens@uza.be; 9 Department of Physical Medicine and Physiotherapy, University Hospital Brussels, 1090 Brussels, Belgium

**Keywords:** autonomic nervous system, autonomic function, electrodermal activity, electrocardiogram, heart rate

## Abstract

Although autonomic nervous system (ANS) dysfunction in Myalgic Encephalomyelitis/Chronic Fatigue Syndrome (ME/CFS) has been proposed, conflicting evidence makes it difficult to draw firm conclusions regarding ANS activity at rest in ME/CFS patients. Although severe exercise intolerance is one of the core features of ME/CFS, little attempts have been made to study ANS responses to physical exercise. Therefore, impairments in ANS activation at rest and following exercise were examined using a case-control study in 20 ME/CFS patients and 20 healthy people. Different autonomous variables, including cardiac, respiratory, and electrodermal responses were assessed at rest and following an acute exercise bout. At rest, parameters in the time-domain represented normal autonomic function in ME/CFS, while frequency-domain parameters indicated the possible presence of diminished (para)sympathetic activation. Reduced parasympathetic reactivation during recovery from exercise was observed in ME/CFS. This is the first study showing reduced parasympathetic reactivation during recovery from physical exercise in ME/CFS. Delayed HR recovery and/or a reduced HRV as seen in ME/CFS have been associated with poor disease prognosis, high risk for adverse cardiac events, and morbidity in other pathologies, implying that future studies should examine whether this is also the case in ME/CFS and how to safely improve HR recovery in this population.

## 1. Introduction

Myalgic Encephalomyelitis/Chronic Fatigue Syndrome (ME/CFS) is a debilitating complex disorder characterized by extreme fatigue and pain complaints [1]. As fatigue and pain are often correlated to symptoms of autonomic dysfunction, involvement of the autonomic nervous system (ANS) has been proposed [2,3]. Two recent systematic reviews examining the existing evidence in ME/CFS have emphasized that controversial findings have been reported and that not all parameters of autonomic function have been studied extensively in this disorder [4,5]. As a consequence, it has been difficult to draw firm conclusions regarding ANS activity at rest in ME/CFS.

Furthermore, little attempts have been made to study ANS activation in response to physical exercise, which is remarkable, as severe exercise intolerance is one of the core features of ME/CFS. More specifically, these patients show decreased cerebral oxygen and blood volume/flow, decreased pain thresholds, impaired oxygen delivery to muscles, elevated levels of oxidative stress and complement proteins, delayed recovery of peripheral muscle fatigue, and symptom exacerbations in response to/during exercise [6]. The impaired cardiodynamic responses to exercise that have been reported in ME/CFS include a slow acceleration of heart rate (HR) and decreased maximum HR during incremental exercise and diminished HR and blood pressure (BP) responses during isometric handgrip exercise [7,8,9,10,11,12]. While heart rate variability (HRV) analysis is the most commonly used measure for the evaluation of cardiac autonomic function at rest and during exercise, studies in ME/CFS have been limited to HR (in beats/minute) and BP responses to physical acute exercise.

Moreover, to date, no studies have examined whether ME/CFS patients have normal autonomic activation during exercise recovery. Yet the ANS does not only play a crucial role in the cardiovascular response to acute exercise, it is also implicated in the recovery from exercise when the balance between the sympathetic and parasympathetic activity needs to be restored [13]. Furthermore, HR recovery after exercise has recently been shown to predict all-cause and cardiovascular mortality as well as sudden death [14,15,16,17].

Therefore, the objective of this study was to assess autonomic function in patients with ME/CFS at rest, during an acute exercise bout, and during recovery from this exercise bout. During these conditions, different autonomous variables, including cardiac, respiratory, and electrodermal responses, were studied concomitantly and were compared to the responses of a healthy control group.

## 2. Materials and Methods

### 2.1. Ethical Approval

This study was designed as a blinded case-control study in line with the STROBE Statement (http://www.strobe-statement.org/) and conducted in accordance with the Declaration of Helsinki with the protocol being approved by the Ethics Committee of the University Hospital Brussels/Vrije Universiteit Brussel (BUN 143201316368). The study was conducted at the department of human physiology from the Vrije Universiteit Brussel, and all participants provided written informed consent prior to study initiation. The abstract of conference presentation of this study has been published [18].

### 2.2. Subjects

Twenty ME/CFS patients and 20 healthy sedentary controls participated in this study. Patients were diagnosed according to the CDCP criteria for ME/CFS [19]. Healthy subjects with a medical history of endocrine abnormalities or diseases known to affect the function of the cardiovascular, immune, or autonomic system were excluded. Sedentary was defined as having a seated occupation and performing ≤3 h of moderate physical activity/week [20]. Moderate activities correspond to activities demanding at least threefold the energy spent passively [20].

All subjects were between 18 and 65 years of age and female as pooling of gender data forms an important source of bias in studies examining exercise physiology and as ME/CFS is predominant in females [21,22]. In order to preclude other confounding factors, subjects were excluded when pregnant, lactating, or <1 year postnatal.

ME/CFS patients were recruited from the department of internal medicine at a university hospital and from a private practice for internal medicine, where co-authors GM and LL respectively checked which patients fulfilled the inclusion criteria and informed them of the study and the possibility of participating in the study. Patients were voluntarily able to decide whether they were willing to participate, without this choice having any effect on their health care. The healthy subjects were recruited amongst healthy friends and relatives from the ME/CFS patients and volunteers who replied to advertisements.

### 2.3. Procedure

During the 1st visit study, one of the researchers (JVO) examined whether the included ME/CFS patients also fulfilled the more recent Canadian criteria for ME/CFS [1], which was the case for all patients. Sociodemographic and disease-related information was collected via a self-composed questionnaire. In order to prevent stress on the day of the assessment each subject was guided through the lab, the different assessment methods and materials were shown, and the full test procedure was explained. The 2nd visit took place within 7–21 days following the 1st visit.

During the 2nd visit, participants performed a submaximal bicycle exercise test with continuous cardiorespiratory monitoring. The Aerobic Power Index test [23,24,25] was performed as described in our previous study [26]. In summary, the exercise protocol commenced at 25 W, and the workload (W) was linearly increased by 25 W/minute, maintaining a cycling rate of 70 rotations/minute until 75% of the age-predicted target HR was reached. The exercise test was concluded by a short cooling down of 30 s, during which the subject kept cycling against a resistance of 25 W, to prevent venous pooling.

**Figure 1:**
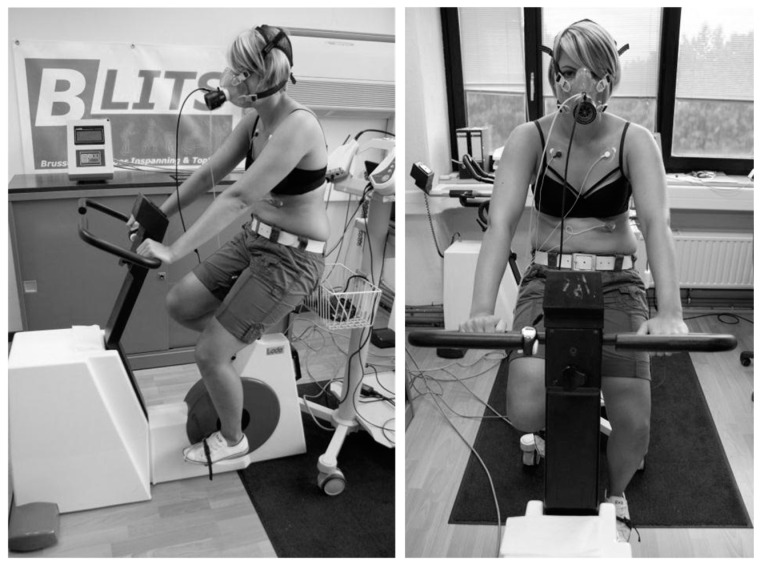
Set-up of the standardized submaximal bicycle exercise test.

A portable cardiopulmonary indirect breath-by-breath calorimetry system (MetaMax 3B, Cortex Biophysik GmbH, Leipzig, Germany) was used to analyze the expired air for ventilatory and metabolic variables. HR during exercise was recorded using ECG electrodes allowing real-time determination of achieved target HR and post-determination of the mean and peak HR during exercise. Immediately following the exercise, subjects were asked to assess their perceived exertion using the Ratings of Perceived Exertion (RPE) Borg scale. The set-up of the exercise test is shown in Figure 1. Before the exercise test (at rest), during the exercise test, and during the subsequent passive recovery period, physiological measures of autonomic function were performed.

All assessments took place in a quiet room with constant ambient temperature (21–23 °C). Subjects were asked to refrain from consuming caffeine, alcohol, nicotine, and physical exertion on the day of the experiment. If medically permissible, medication acting on (1) the cardiovascular system was withheld on the day of the examinations, as this type of medication can prevent achievement of the target HR during the exercise test; and (2) the central nervous or hormonal systems was withheld for at least 48 h before the examinations took place, as these types of medications can influence autonomic function. Subjects were asked to report whether they complied with these instructions.

### 2.4. Physiological Measures of Autonomic Function

**Figure 2:**
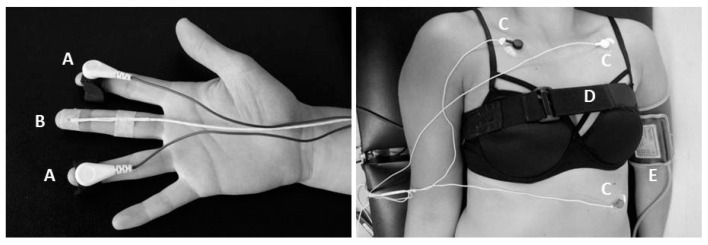
Electrode placement. Legends: A: skin conductance sensors, B: skin temperature sensor, C: ECG electrodes standard lead II placement, D: elastic belt with piezoelectric sensor to measure respiration rate, E: inflatable cuff placement of the electronic blood pressure monitor.

The Nexus-10 wireless and portable telemetry data acquisition system (Mind Media BV, Roermond-Herten, The Netherlands) was used to physiologically assess autonomic responses such as skin conductance (SC), skin temperature (ST), electrocardiogram (ECG), and respiration rate (RR). Blood pressure (BP) was measured using an electronic blood pressure monitor. Placement of the sensors is presented in Figure 2. Measures were taken continuously during 10 min of rest before and following the bicycle exercise; the latter was considered as the recovery period. During the measurements at rest and recovery, the subject lay supine with the forearms in supination beside the body and was asked not to talk, move, or close the eyes. Measures during exercise were limited to ECG. Signals were analyzed offline with the BioSig toolbox in MATLAB software (MathWorks, Natick, MA, USA). For each measurement, the overall mean across the recording periods was calculated (mean PRE, mean DURING, mean POST) (Guideline on heart rate variability, 1996).

HRV was assessed through calculation of the root mean square of successive differences between NN intervals (RMSSD) and frequency analysis performed using the quotient (LF/HFratio) of low-frequency components (i.e., the power in the low-frequency (LF) range between 0.04 and 0.15 Hz) over high-frequency components (i.e., the power in the high-frequency (HF) range between 0.15 and 0.40 Hz) after fast Fourier transformation [27,28]. RMSSD reflects the integrity of vagus nerve-mediated autonomic control of the heart [29]. The LF/HF ratio is an indicator of cardiac sympathetic modulation and sympatho/vagal balance [28]. The efferent vagal activity is a major contributor to the HF component, while LF is mediated by both sympathetic and parasympathetic modulations. SC, a parameter of peripheral sympathetic activity, was assessed by extracting a measurement of the (tonic) background level i.e., skin conductance level (SCL), and of the time-varying (phasic) responses i.e., skin conductance responses (SCR) [30]. Changes in ST as small as 0.001 °C in a range of 10–40 °C were recorded. Peak detection was applied on the respiration data, and the number of peaks/minute reflected the RR. BP was measured at the start and at the end of the 10-min periods preceding and following the bicycle exercise.

### 2.5. Statistics

Data analysis was performed using SPSS 20.0. Descriptives were calculated, and the normality of the data was evaluated using the Shapiro–Wilk test and visual assessment of histograms, QQ-plots, and boxplots. When possible outliers were identified during this assessment, it was examined whether these were in the normal range of the according measures or whether they were considered as outliers using the outlier labeling rule [31].

Comparability of the groups at baseline and regarding exercise related outcome was evaluated using the Independent Samples *t*-test or Mann–Whitney U test depending on the distribution of the data. The Fisher exact test or the Pearson Chi-Square test were used to analyze binary and categorical data.

Not all outcome measures of autonomic function were normally distributed, and as logarithmically transformation did not resolve this issue for all parameters, further analysis was performed using univariate analyses. For each group (ME⁄CFS and CON), possible differences in the response of the outcome measures to exercise (PRE vs. DURING vs. POST) was examined using either the Paired Samples *t*-test or the Wilcoxon Signed Rank test. In case a significant difference was found regarding the recovery (PRE vs. POST), the 10-min baseline and recovery periods were additionally divided into five equal 2-min long periods to examine the course of the autonomic responses over time. The difference in exercise response between the two groups regarding autonomic function was examined using the Mann–Whitney U or Independent Samples *t*-testing.

The significance level was set at *p* < 0.05.

Since no studies had examined autonomic nervous function during/following physical exercise in ME/CFS before, no data were available to provide a basis for the a priori power analysis. Therefore, the sample size was based on a similar study [32] which evaluated autonomic dysfunction based on HRV parameters in time and frequency domains and HR recovery in response to a submaximal bicycle exercise test in females with chronic stroke on the one hand, and on a study [26] that used a submaximal bicycle exercise test to evaluate exercise intolerance in females with ME/CFS on the other hand. The calculations revealed that 16 to 21 subjects/group were required to obtain a power of 0.80 with α = 0.05.

## 3. Results

### 3.1. Subjects

The sociodemographic data are shown in Table 1, and no significant group differences were found. Even though subjects were asked to refrain from central acting medication on the day of exercise testing, six ME/CFS and one control subject reported using medication (between-group *p* = 0.091). Only two ME/CFS patients took central acting selective serotonin reuptake inhibitors, while all other subjects took peripheral acting drugs including paracetamol, diclofenac, and non-steroid anti-inflammatory drugs.

### 3.2. Exercise-Related Outcomes

All subjects were able to complete the exercise test. There were no significant between-group differences regarding theoretical target HR, actual achieved peak HR and mean HR, cycling time, maximum workload achieved, and exercise capacity, as can been seen in Table 2. Although both groups performed a similar exercise test and showed similar exercise capacity, the exercise was perceived as heavier and more strenuous by the ME/CFS patients (*p* < 0.001).

### 3.3. Autonomic Function

**Figure 3:**
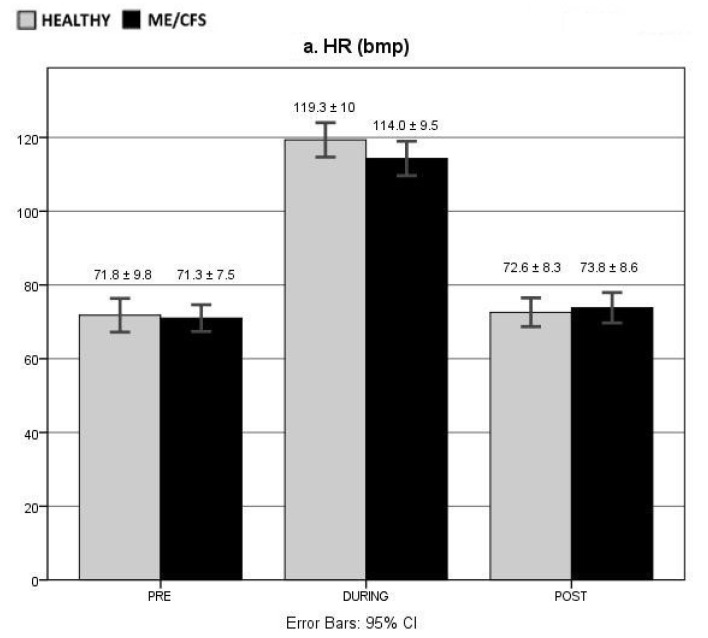

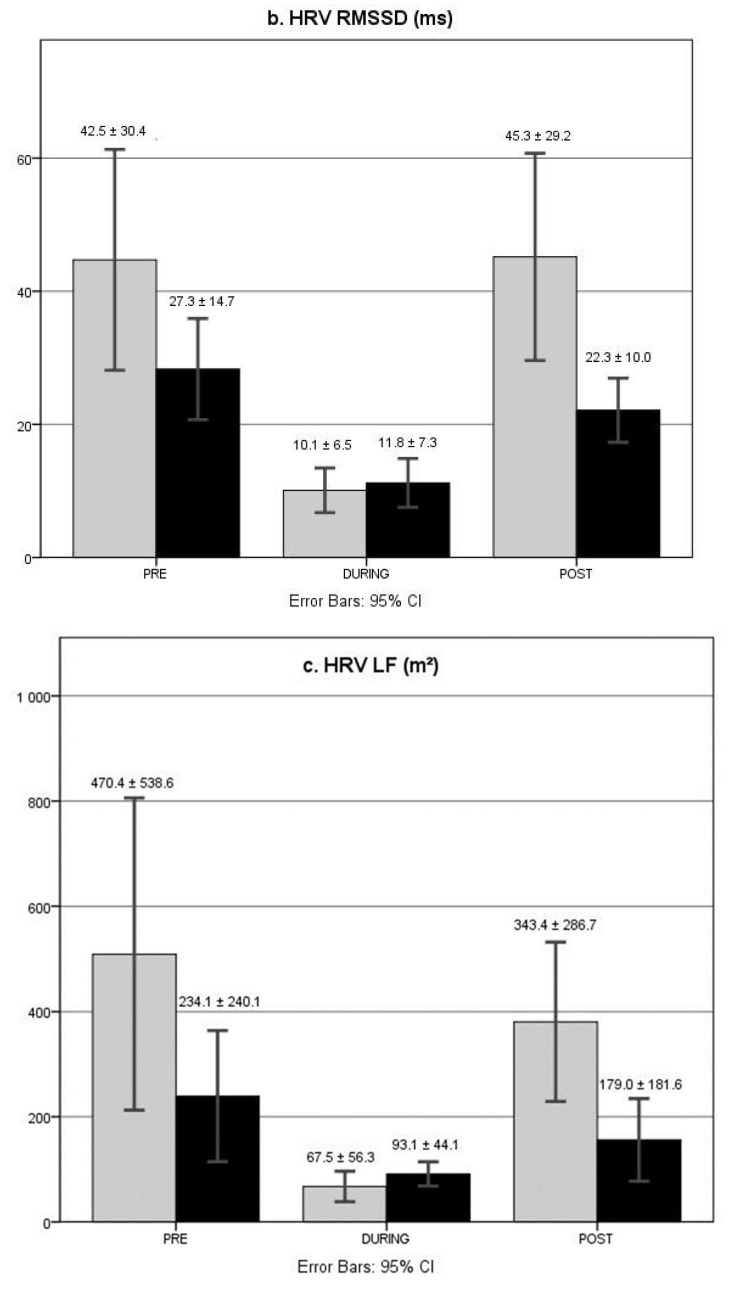

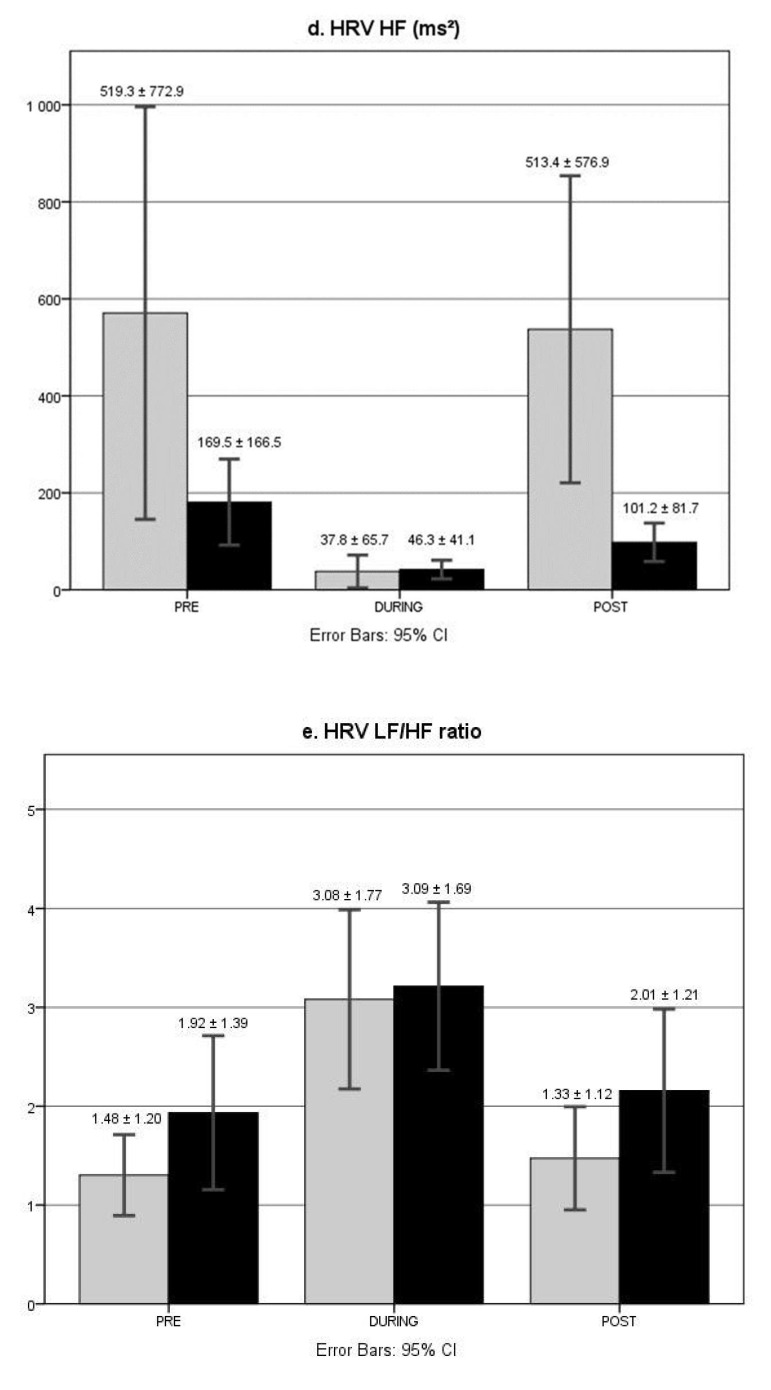

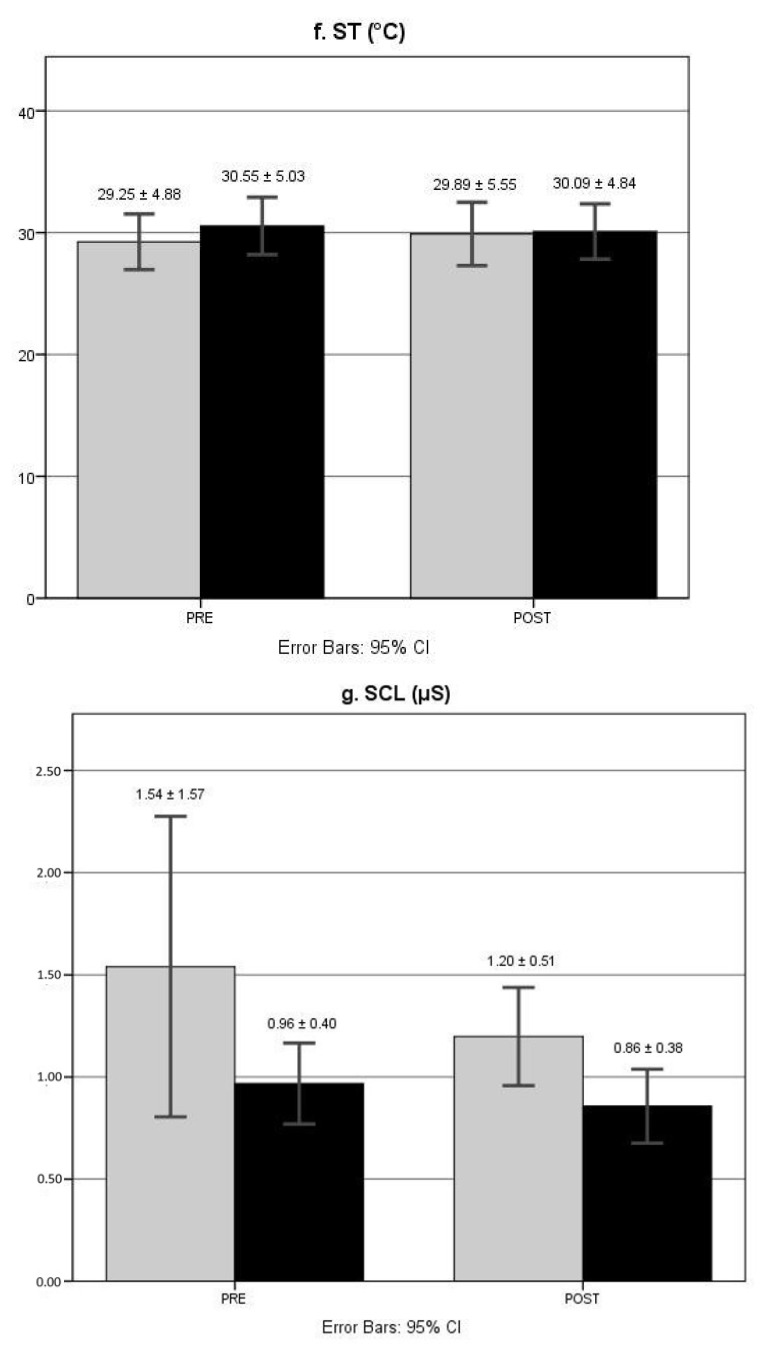

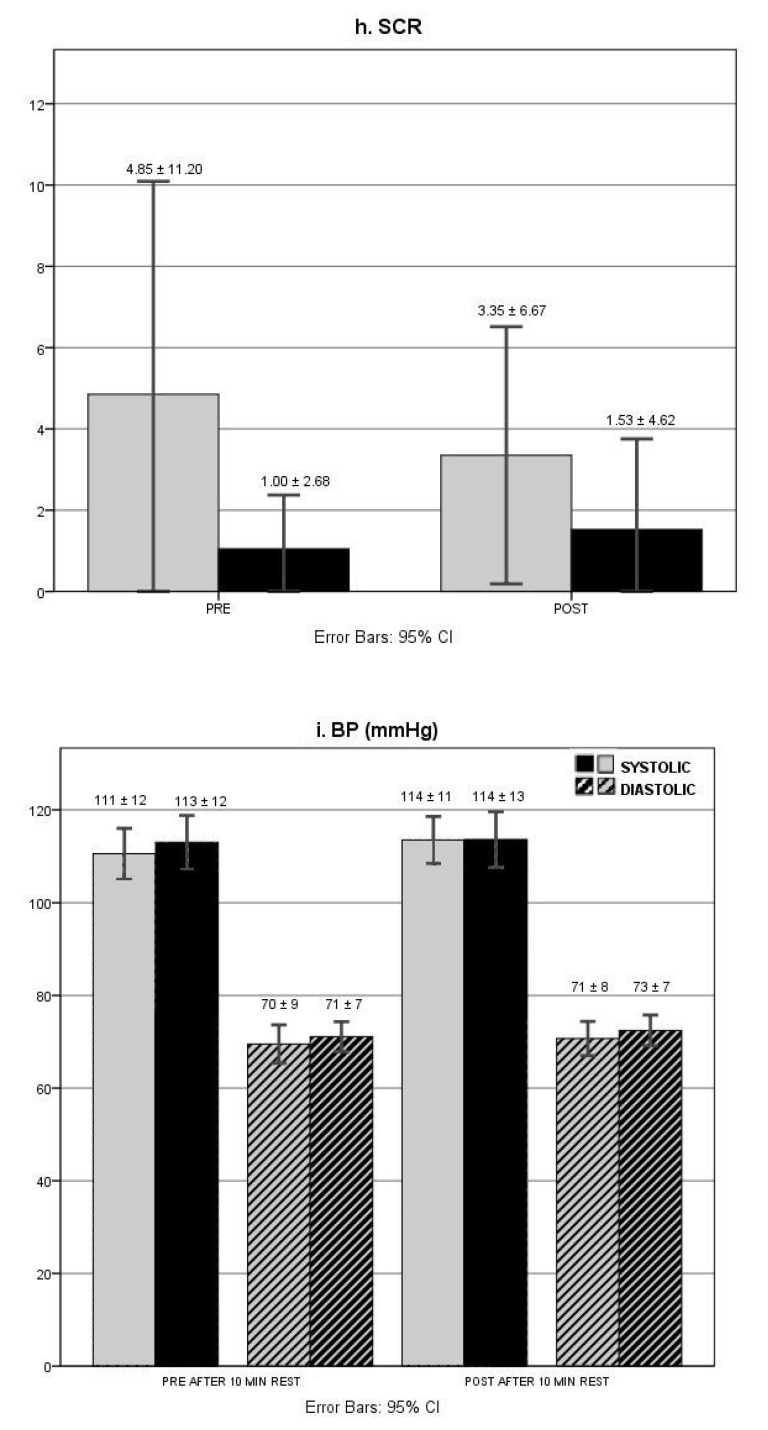

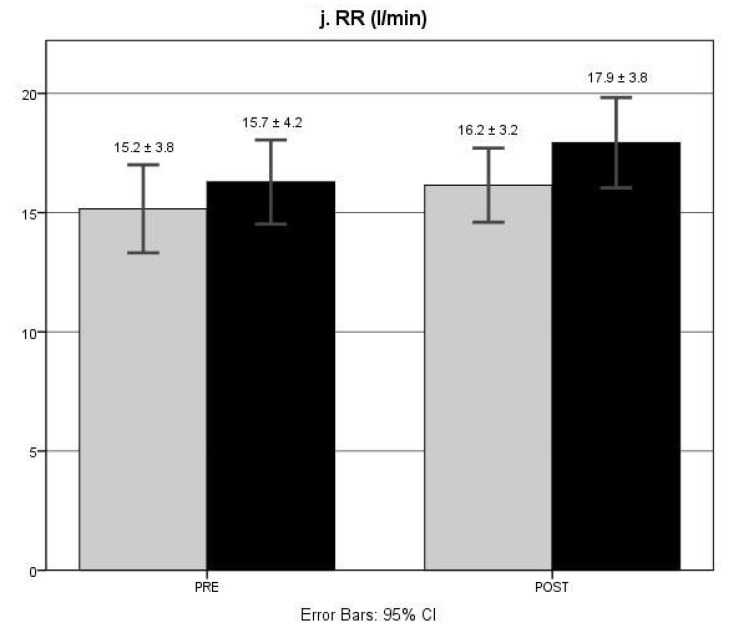
Means and standard deviations of autonomic outcome measures. The specific outcome measure each figure refers to is in depected in the figure itself. (**a**) HR (bpm); (**b**) HRV RMSSD (ms); (**c**) HRV LF (m^2^); (**d**) HRV HF (ms^2^); (**e**) The LF/HF ratio; (**f**) ST (°C); (**g**) SCL (μS); (**h**) SCR; (**i**) PB (mmHg); (**j**) RR (I/min). Legends: The *y*-axis represents the quantification of the measure, the *x*-axis represents the time point of the measure (PRE: measure taken prior to exercise, DURING: measure taken during exercise, POST: measure taken post-exercise). Gray bars: healthy controls, black bars: ME/CFS. Abbreviations: BP: blood pressure, bpm: beat per minute, CI: confidence interval, HR: high frequency, HR: heart rate, HRV: heart rate variability, LF: low frequency, ME/CFS: Myalgic Encephalomyelitis/Chronic Fatigue Syndrome, RMSSD: root mean square of successive differences between NN intervals, RR: respiration rate, SCL: skin conductance level, SCR: skin conductance response, ST: skin temperature.

The mean PRE, DURING, and POST values for each group are presented in Figure 3; values of the 2-min intervals are presented in the supporting information (S1).

### 3.4. HR

No between-group differences were found for mean HR at baseline (PRE *p* = 0.870), during exercise (DURING *p* = 0.092), and during recovery (POST *p* = 0.655) (Figure 3a). During exercise, the mean HR was higher than at rest in both groups (PRE vs. DURING *p* < 0.001). After the exercise, the mean HR declined in both groups (DURING vs. POST *p* < 0.001), but a differential response was seen regarding full recovery. The controls showed no significant differences between HR measured during recovery and HR at rest (PRE vs. POST *p* = 0.578), indicating a quick recovery to the original baseline levels following exercise. In ME/CFS, this was not the case, as a significantly higher HR was observed during recovery than at rest (PRE vs. POST *p* = 0.031), and at the end of the 10-min recovery, the HR remained above the baseline levels (PRE8-10 min vs. POST8-10 min *p* = 0.020), which was not the case for the controls.

### 3.5. HRV

No significant group differences were found regarding RMSSD at baseline (PRE *p* = 0.060) (Figure 3b). In both groups, a similar response to exercise was seen (DURING *p* = 0.613), with RMSSD decreasing (PRE vs. DURING ME/CFS *p* = 0.003, CON *p* < 0.001). After exercise, RMSSD values increased again (DURING vs. POST ME/CFS *p* = 0.006, CON *p* < 0.001), but RMSSD during recovery did differ significantly between groups. ME/CFS subjects showed lower values than the controls over the whole recovery period (POST *p* = 0.010) as well as at the different time intervals (*p* between 0.003 and 0.041). The overall RMSSD response during recovery was similar to baseline in both groups (PRE vs. POST ME/CFS *p* = 0.059, CON *p* = 0.881).

Both HF and LF were significantly lower in ME/CFS than in the controls at baseline (PRE HF *p* = 0.024, LF *p* = 0.038) and during recovery (POST HF *p* = 0.001, LF *p* = 0.015) (Figure 3c,d). During exercise, this difference between groups dissipated for HF (*p* = 0.245) but was sustained for LF (*p* = 0.029), and HF (PRE vs. DURING ME/CFS *p* = 0.014, CON *p* < 0.001) as well as LF (PRE vs. DURING ME/CFS = 0.022, CON *p* < 0.001) levels decreased in the two groups. In the controls, both HF and LF increased during recovery (DURING vs. POST respectively *p* = 0.022 and *p* < 0.001). While HF significantly increased during recovery in the ME/CFS patients, the increase in LF was not significant (DURING vs. POST respectively *p* = 0.016 and *p* = 0.193). The HF of the controls during exercise recovery was similar as in baseline (PRE vs. POST *p* = 0.709), while their LF was significantly lower during the recovery period (PRE vs. POST *p* = 0.012). In ME/CFS, the opposite effect was observed, where LF during recovery was similar to baseline (PRE vs. POST *p* = 0.126), and HF was significantly decreased during the whole recovery period (PRE vs. POST *p* = 0.044) and at the end of the 10-min recovery period (PRE8-10 min vs. POST8-10 min *p* = 0.016).

The LF/HF ratio was similar between groups at baseline (PRE *p* = 0.314) and during exercise (DURING *p* = 0.961) but higher in ME/CFS than in the controls during recovery (POST *p* = 0.035) (Figure 3e). At the end of the recovery period, the group difference was no longer present (POST8–10 min *p* = 0.057). While the LF/HF ratio increased significantly from rest to exercise in controls, this was not the case in the ME/CFS group (PRE vs. DURING ME/CFS *p* = 0.078, CON *p* = 0.001). However, values decreased in both groups during the post-exercise recovery period (DURING vs. POST ME/CFS *p* = 0.009, CON *p* = 0.004) until they were no longer significantly different from baseline (PRE vs. POST ME/CFS *p* = 0.841, CON *p* = 0.502).

### 3.6. Electrodermal Responses

SCL were lower in ME/CFS than in the controls, but only during recovery did this difference reach significance (PRE *p* = 0.165, POST *p* = 0.016) (Figure 3g). The group difference was observed throughout the whole recovery period (POST intervals between 0.018 and 0.044). Although SCL were lower during recovery than at baseline, the mean difference was not significant in either group (PRE vs. POST ME/CFS *p* = 0.184, CON *p* = 0.351). SCR at baseline and during recovery were not significantly different from each other (PRE vs. POST ME/CFS *p* = 0.916, CON *p* = 0.575) or between the two groups (PRE *p* = 0.758, POST *p* = 0.569, POST intervals *p* between 0.309 and 0.835) (Figure 3h and Figure S1).

ST during recovery did not significantly differ from the baseline value in neither group (PRE vs. POST ME/CFS *p* = 0.135, CON *p* = 0.823), and no group differences were observed (PRE *p* = 0.383, POST *p* = 0.820) (Figure 3f).

### 3.7. RR

RR was similar between groups at baseline (PRE *p* = 0.656) (Figure 3j). While RR was not measured during exercise, similar between-group ventilatory outcomes were shown from the ergospirometric measures (cfr. 3.2). At the start of the recovery period, the ME/CFS group had a higher RR than controls (POST1-2 min *p* = 0.032), their RR decreased in the following 8 min of recovery returning to similar values as in the control group (POST *p* = 0.343, POST3–4,5–6,7–8,8–10 min *p* between 0.155 and 0.851). However, throughout the recovery period, RR remained higher than at baseline for both groups (PRE vs. POST ME/CFS *p* = 0.003, CON *p* = 0.005).

### 3.8. BP

BP values at the start and at the end of 10 min of supine resting were similar (*p* between 0.094 and 0.617), and there were no group differences (*p* between 0.437 and 0.528) (Figure 3i). Both groups responded in the same way to the exercise test (systolic BP *p* = 0.589, diastolic BP *p* = 0.588), with systolic BP increasing (ME/CFS *p* = 0.001, CON *p* = 0.003) while diastolic BP remained stable (ME/CFS *p* = 0.262, CON *p* = 0.275). After 10 min of supine recovery, both systolic and diastolic BP were similar to the values seen at rest (*p* between 0.063 and 0.767) and between groups (systolic BP *p* = 0.979, diastolic BP *p* = 0.467).

## 4. Discussion

This study assessed autonomic function in patients with ME/CFS at rest, during an acute bout of physical exercise, and during exercise recovery. HRV frequency-domain parameters indicated the possible presence of diminished cardiac (para)sympathetic activation at supine rest, while blood pressure, respiratory, electrodermal, and HRV parameters in the time-domain represented normal autonomic function at rest in ME/CFS. A similar (para)sympathetic modulation took place during exercise in ME/CFS as in healthy people; however, the magnitude of this modulation was impaired in those with ME/CFS. Reduced parasympathetic reactivation during recovery from exercise was observed in ME/CFS.

### 4.1. Autonomic Function at Rest in ME/CFS

In the present study HR, BP, RMSSD, RR, SCL, SCR, and ST suggest normal autonomic activity during supine lying in ME/CFS. A similar amount of studies exist that confirm or refute the presence of a differential HR and BP in ME/CFS at rest (reviewed in [4,5]). Our findings showed that ME/CFS patients have a similar resting HR and systolic/diastolic BP as healthy sedentary subjects. This was also the case for RMSSD, which is in line with previous observations (reviewed in [4,5]). Although LF and HF in ME/CFS were lower than in healthy subjects, the LF/HR ratio was similar in both groups. This observation could indicate reduced sympathetic and parasympathetic activity in ME/CFS at rest, while the sympatho/vagal balance is maintained. As LF is related to baroreflex function, a decreased LF could reflect baroreflex failure, which in turn is often observed in case of cardiac sympathetic denervation [33]. However, further research using beat-to-beat measures is necessary to confirm this assumption.

The current knowledge regarding electrodermal function in ME/CFS is very limited, as only one study [34] has examined this aspect of autonomic function before in this population. The findings from that study suggested that ME/CFS patients have normal SCR but reduced SCL and increased ST. Our findings could not confirm the latter observations. Although mean SCL were lower and mean ST was higher in ME/CFS than in the healthy group, the difference was not significant, and the mean values were lower than those reported by Pazderka-Robinson et al. [34].

### 4.2. Autonomic Function during an Acute Aerobic Exercise Bout in ME/CFS

Cardiac responses were studied during the performance of a submaximal, incremental aerobic exercise test on a cycle ergometer. Performance parameters such as the ability to complete the exercise protocol, exercise capacity, final power output, and cycled time were similar between ME/CFS and healthy subjects, which is in line with previous reports [26,35] and suggests equal demands were required from the ANS during exercise in both populations. In normal circumstances, exercise is accompanied with dynamic changes in cardiac responses, which results in an increased blood flow and redistribution of the blood to satisfy the energy demands of the working muscles. While systolic BP will increase during exercise, diastolic BP remains relatively constant. HR increases immediately at the onset of activity as a result of parasympathetic withdrawal [36]. As exercise continues, further increases in HR are due to the action of the sympathetic nervous system. The increased sympathetic nervous activity is reflected in an increased LF/HF ratio and has been described to occur when HR exceeds 100 bpm [37,38].

Our findings in ME/CFS are in line with these observations in healthy people. The BP responses during exercise were normal in ME/CFS, with systolic BP increasing while diastolic BP remained stable. The subjects’ mean HR increased during exercise testing while the mean HF dropped, which can be interpreted as a decrease in parasympathetic modulation. As this was observed in both ME/CFS and healthy subjects, we can conclude that this autonomic mechanism functions normally in ME/CFS. Since sympathetic activity cannot be easily isolated from LF, the LF/HR ratio is a more adequate parameter to provide us with insights regarding sympathetic modulation and sympatho/vagal balance during the exercise test [28]. The LF/HF ratio increased in the controls, reflecting sympathetic dominance and parasympathetic inhibition during exercise. Although the mean LF/HF ratio also increased in ME/CFS in response to the exercise, the decrease was not large enough to reach significance. The latter observation might indicate that although a similar autonomic modulation seems to take place during exercise in ME/CFS as in healthy people, the magnitude of this modulation might be impaired in ME/CFS. Further research in larger sample sizes is warranted to confirm these assumptions.

### 4.3. Autonomic Function during Recovery from Exercise in ME/CFS

Autonomic activity was assessed during a 10-min passive recovery period following the aerobic exercise test. HR and BP responses during recovery were similar for both groups, although those with ME/CFS did not manage to fully restore their elevated HR to rest levels as healthy subjects did. It has been shown that a delayed HR recovery, which is the return of the HR during post-exercise recovery to the pre-exercise HR by parasympathetic reactivation, is an independent predictor of overall mortality and may be linked to adverse prognosis [14,15,16,17]. Therefore, the lack of HR recovery observed during the two first minutes of the passive recovery period and the delayed HR recovery observed over the full 10 min of the recovery period could have important implications, and this should be further examined. Specifically, future studies should attempt to evaluate HR recovery during the first or second minute after immediate cessations of the acute exercise bout (i.e., passive recovery) or during cooling down (i.e., active recovery) [39]. As aerobic endurance training has been shown to accelerate HR recovery after exercise in healthy people [40]), future studies are required to determine whether this type of training can also improve HR recovery in ME/CFS and if this can be performed without inducing symptom exacerbations [26].

Although RMSSD, LF, and HF evolved the same way in ME/CFS as in healthy subjects, again, the magnitude of these modulations was smaller in ME/CFS. More specifically, in ME/CFS, the increases of RMSSD and LF during recovery from exercise were reduced, and although a similar increase was seen for HF, HF did not manage to restore to pre-exercise levels. The latter observations indicate that ME/CFS patients manage to restore their HRV following exercise, but that the magnitude of their HRV following exercise is lower than in healthy people. The inability to restore HF to pre-exercise levels in ME/CFS suggest a reduced parasympathetic modulation during recovery from exercise in these patients.

It is generally agreed that there is parasympathetic withdrawal and sympathetic excitation during exercise and that these effects are reversed in recovery [36]). Hence, the LF/HF ratio will decrease during recovery. This was the case for both ME/CFS patients and healthy subjects, and the LF/HF reached similar values as at rest. Although the recovery of LF/HF took place in ME/CFS, the magnitude was smaller, and more time (8 min) was necessary to fully restore HF/LF as healthy people did. While both groups showed equal LF/HF ratios at baseline, during recovery, ME/CFS patients had higher LF/HF than healthy subjects, suggesting a dysfunctional balance between the parasympathetic and sympathetic nervous system following recovery. However, LF/HF was restored at the end of the 10-min recovery period, which possibly indicates a delayed recovery in ME/CFS.

It has been suggested that the HF or parasympathetic tone represents an individual’s ‘functional capacity’ for exercise [41]. Our HRV results in ME/CFS demonstrate a reduced functional capacity for exercise (decreased HF power at rest). Since physical training has been shown to cause an increase in parasympathetic tone [42], it could be beneficial for ME/CFS. Nonetheless, the training intensity should be kept within the limits of the individual’s capacity in order to not worsen the already present autonomic imbalance; yet, it needs to be high enough to invoke a training effect. The balance between accurate training stimuli and recovery is necessary to avoid post-exertional malaise. Since each training session causes an acute decrease in parasympathetic activity, enough rest is required to rebound back toward (and beyond) the original pre-training level. Hautala et al. [41] have suggested to use the HF power obtained by HRV analysis as guidance in determining the correct training volume. On days when decreased parasympathetic activity is observed in the morning, expressing insufficient recovery from the previous exercise, a lower training load or rest is prescribed; and conversely, on days with high parasympathetic activity, a higher training load is allowed. Unfortunately, there is currently little knowledge regarding the best exercise intensity for improving autonomic balance in individuals with a dysfunctional stress system.

Similar responses in RR were seen in both groups, with RR remaining above baseline levels during the 10 min of recovery. In the 2 first minutes of recovery, ME/CFS had higher RR than healthy people, but after 2 min, the RR of the patients had decreased to similar levels as the healthy group. Peripheral autonomic activity was studied by examining SC and ST during the recovery period. SCL, which were similar between groups at rest and showed an analogous evolution during recovery, were lower in the ME/CFS patients compared to the healthy subjects throughout the recovery period. While the difference in mean SCL did not seem to increase during the recovery period compared to baseline, this difference between the groups seems to be the consequence of the diminished SCL variability in the control group during the recovery period. SCR responses during recovery were similar as at rest, and ME/CFS patients showed the same reactions as healthy people. Currently, there is no literature available regarding electrodermal responses during exercise recovery in ME/CFS, but our findings indicate that overall, these responses are similar as in healthy people.

### 4.4. Strengths and Limitations

The results should be interpreted light of the following study limitations. As not all subjects were examined at the same time of the day, and not all subjects complied with instructions regarding the wash-out period of medication, we cannot exclude the possibility that this influenced the results. As only women were studied, care should be taken with the extrapolation of these results to the male ME⁄CFS population. As the study was performed at the Human Physiology lab and participants had to perform an exercise test, it is obvious that only patients with ME/CFS with mild to moderate disease severity participated in this study. When interpreting the study results, one should keep in mind that although post-exercise assessments were taken as quickly as possible, subjects needed to reposition themselves from the bicycle to supine position and finger sensors needed to be reattached before the assessments were started. In addition, using this protocol, it was not possible to evaluate respiratory measures during exercise in the same way as at rest or recovery, or to examine the electrodermal responses during exercise. Furthermore, the sample size was based on primary outcomes of interest, namely, HRV parameters in time and frequency domains and HR recovery, and thus, it cannot be excluded that for the other outcome variables that were studied, the sample size was too low in order to draw firm conclusions.

The study has several strengths by complying with previous recommendations regarding research in ME/CFS and preventing confounding factors. Patients fulfilled the diagnostic criteria for ME/CFS described by Fukuda et al. in 1994 [19] as well as the more recent Canadian criteria described by Carruthers et al. in 2011 [1]. As previously suggested, measures of cardiac, respiratory, and electrodermal activity were performed to study different aspects of the ANS [5]. Sedentary healthy subjects were included and showed similar exercise capacity levels and performance parameters as the ME/CFS group, which suggests that deconditioning was not primarily responsible for the observed group differences. A submaximal exercise protocol that is reliable and valid for testing these populations was used [23,24,25]. Finally, all measures were undertaken in a standardized way and in a temperature-controlled environment.

## 5. Conclusions

The findings of this study suggest reduced autonomic modulation during exercise/reactivation during exercise recovery in ME/CFS. As delayed HR recovery and/or a reduced HRV implicate a poor disease prognosis and have been associated with higher risk for cardiac events and morbidity, further studies on methods to improve HR recovery in a safe way in ME/CFS are warranted. This mainly implies improving parasympathetic reactivation following physical exercise and providing sufficient long recovery periods following exercise.

## Figures and Tables

**Table 1:** Demographic baseline characteristics.

	ME/CFS Group (*n* = 20)	Healthy Group (*n* = 20)	Between-Group Comparison (*p*-Value)
**Age, years**			0.155
Mean (SD)	41.6 (9.8)	34.6 (15.2)	
**Length, cm**			0.935
Mean (SD)	168 (5)	168 (8)	
**Weight, kg**			0.168
Mean (SD)	68.1 (14.9)	73.9 (15.6)	
**Handedness**			1.000
Right (*n*)	17	16	
Left (*n*)	3	4	
**Employment status**			0.208
Student (*n*)	1	6	
Retired (*n*)	0	1	
Full-time (*n*)	4	2	
Part-time (*n*)	6	4	
Non-employed (*n*)	9	7	
**Years of education**			0.177
Mean (SD)	14.4 (2.8)	15.6 (2.7)	
**Highest degree of education**			0.054
Primary school (*n*)			
Secondary education (*n*)	9	12	
Higher education—university or college (*n*)	2	1	
Higher education—adult education social	8	7	
advancement course (*n*)	1	0	
**Marital status**			0.609
Single (*n*)	9	12	
Living together (*n*)	2	1	
Married (*n*)	8	7	
Widow (*n*)	1	0	
**Children**			
Yes	7	12	0.205
No	13	8	
Mean number (SD)	1 (1.0)	1 (1.3)	0.640
**Time from diagnosis, months**			
Mean (SD)	70.3 (56.8)	NA	NA

Abbreviations: SD: standard deviation, n: number of, NA: not applicable.

**Table 2:** Exercise-related outcomes.

	ME/CFS Group (*n* = 20)	Healthy Group (*n* = 20)	Between-Group Comparison (*p*-Value)
**HR, bpm** Theoretical target HR peak * Actual achieved HR peak Mean HR	134 (7) 140 (9) 114 (10)	140 (12) 142 (10) 119 (10)	0.149 0.453 0.092
**Cycling time, min**	3.86 (1.00)	4.15 (1.15)	0.401
**Peak Workload**	109 (25)	118 (25)	0.327
**VO2 peak, mL/min/kg**	16.98 (4.25)	19.96 (6.80)	0.112
**VE peak, L/min**	31.81 (9.67)	31.61 (11.30)	0.758
**RER peak**	0.76 (0.89)	72 (0.08)	0.101
**RPE**	16 (0.3)	12 (2)	<0.001

Abbreviations: HR: heart rate, VO2 peak: peak oxygen uptake, VE peak: peak ventilation, RER peak: respiratory exchange ratio, RPE: rate of perceived exertion, * corresponds with 75% of the age-predicted target HR.

## Supplementary Materials

The following are available online at https://www.mdpi.com/article/10.3390/jcm10194527/s1, Figure S1: Means and standard deviations of autonomic outcome measures over 2-min intervals.

## Abbreviations

ANS	autonomic nervous system
ECG	electrocardiogram
CFS	chronic fatigue syndrome
HR	heart rate
HRV	heart rate variability
HF	high frequency
LF	low frequency
ME	myalgic encephalomyelitis
RMSSD	root mean square of successive differences between NN intervals
RPE	ratings of perceived exertion
RR	respiration rate
SC	skin conductance
SCL	skin conductance level
SCR	skin conductance responses
ST	skin temperature
W	workload
