# Supplementary figures and images for: Reduced Parasympathetic Reactivation during Recovery from Exercise in Myalgic Encephalomyelitis/Chronic Fatigue Syndrome
Source: J Clin Med. 2021 Sep 30;10(19):4527. doi: 10.3390/jcm10194527 (PMC8509376; doi:10.3390/jcm10194527)

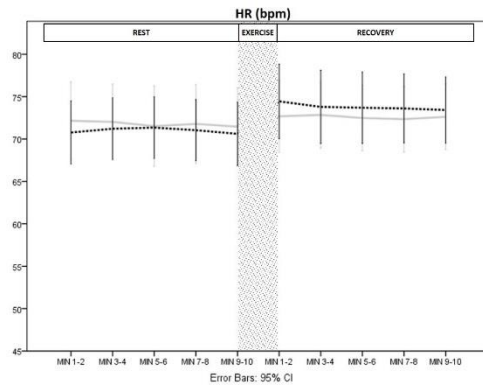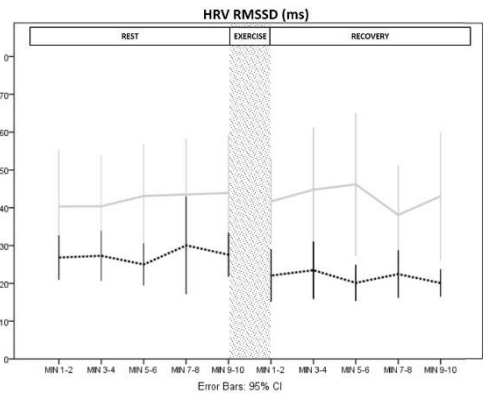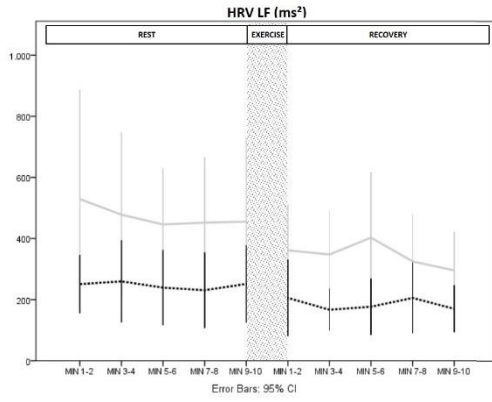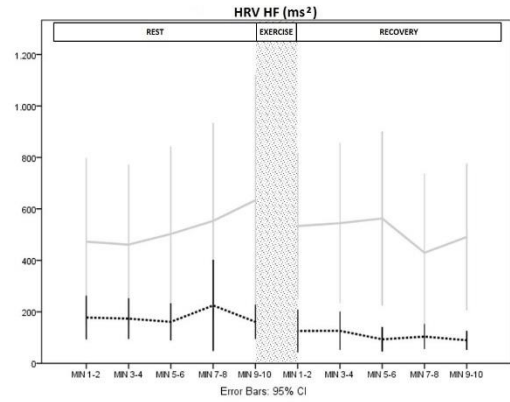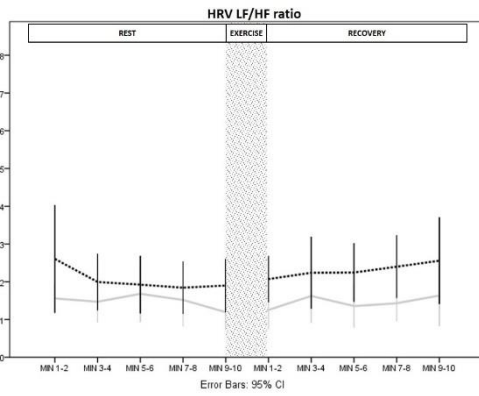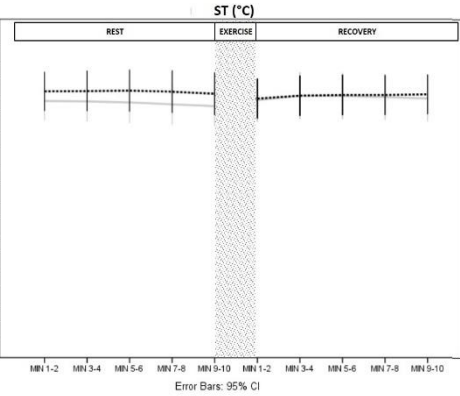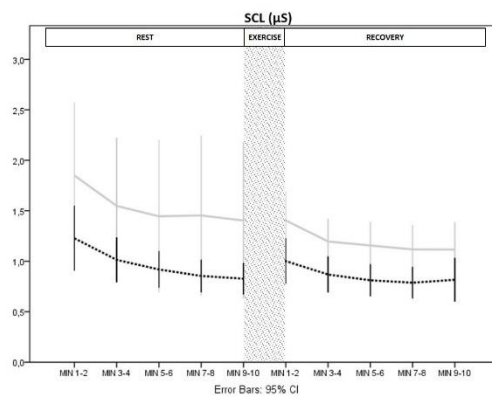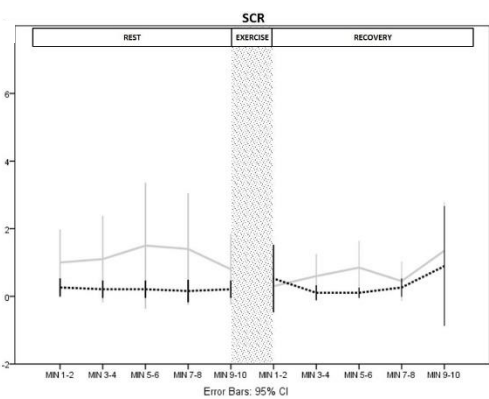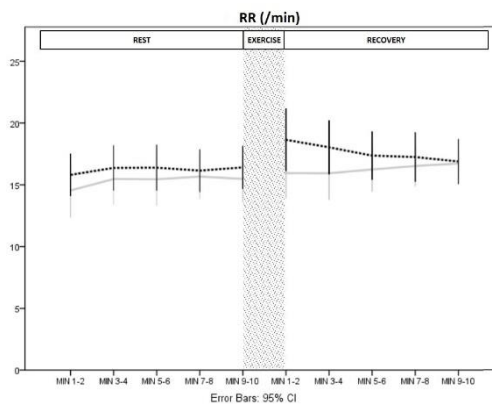

ME/CFS ■

HEALTHY ■

Supplement: Supplementary file 1 [file jcm-10-04527-s001.zip › jcm-1302385-supplementary.pdf]
